# Immunomodulatory activity of pidotimod administered with standard antibiotic therapy in children hospitalized for community-acquired pneumonia

**DOI:** 10.1186/s12967-015-0649-z

**Published:** 2015-09-03

**Authors:** Susanna Esposito, Micaela Garziano, Veronica Rainone, Daria Trabattoni, Mara Biasin, Laura Senatore, Paola Marchisio, Marta Rossi, Nicola Principi, Mario Clerici

**Affiliations:** Pediatric Highly Intensive Care Unit, Department of Pathophysiology and Transplantation, Università degli Studi di Milano, Fondazione IRCCS Ca’ Granda Ospedale Maggiore Policlinico, Via Commenda 9, 20122 Milan, Italy; Immunology Unit, Department of Biomedical and Clinical Sciences, Luigi Sacco Hospital, Università degli Studi di Milano, Milan, Italy; Department of Clinical Sciences and Community Health, Università degli Studi di Milano, Milan, Italy; Department of Physiopathology and Transplantation, Università degli Studi di Milano, Milan, Italy; Don C. Gnocchi Foundation IRCCS, Milan, Italy

**Keywords:** Children, Community-acquired pneumonia, Immunomodulatory activity, Immunostimulants, Pidotimod, Respiratory tract infection

## Abstract

**Background:**

Several attempts to improve immune function in young children have been made and encouraging results have been collected with pidotimod (PDT), a synthetic dipeptide molecule that seems to have immunomodulatory activity on both innate and adaptive responses. Until now, the effects of PDT on the immune system have only been studied in vivo after long-term administration to evaluate whether its immunomodulatory activity might prevent the development of infections. This study was planned to evaluate the immunomodulatory activity of PDT administered together with standard antibiotic therapy in children hospitalized for community-acquired pneumonia (CAP).

**Methods:**

A total of 20 children hospitalized for community-acquired pneumonia (CAP) were randomized at a 1:1 ratio to receive either standard antibiotics plus pidotimod (PDT) or standard antibiotics alone to evaluate the immunomodulatory activity of PDT. Blood samples for the evaluation of immunological parameters were drawn at the time of recruitment (T0) (i.e., before therapy administration), at T3 and T5 (i.e., 3 and 5 days after the initiation of therapy) as well as at T21 (i.e., 7 days after the therapy ended).

**Results:**

Following pneumococcal polysaccharide stimulation, the percentage of dendritic cells (DCs) expressing activation and costimulatory molecules was significantly higher in children receiving PDT plus antibiotics than in the controls. A significant increase in tumor necrosis factor-α and/or interleukin-12 secretion and expression of toll like receptor 2 was observed in PDT-treated children compared with controls; this was followed by an increased release of proinflammatory cytokines by monocytes. In the PDT-treated group, mRNA expression of antimicrobial peptides and genes involved in the inflammatory response were also augmented in comparison with the controls.

**Conclusions:**

These results demonstrate, for the first time, that PDT administered together with standard antibiotics is associated with a favorable persistent immunomodulatory effect in children with CAP.

## Background

In the first years of life, recurrent respiratory tract infections (RRTIs) commonly occur in otherwise healthy children due to an increased risk of exposure to infectious agents coupled with the immaturity of the immune system [1, 2]. Both innate and adaptive immunity develop gradually, and some aspects are not fully functional until 5–7 years of age [3]. In comparison with adults, younger children have lower concentrations in their respiratory mucus of several proteins that exert antibacterial activity (e.g., defensins, lysozyme, IgA) as well as proteins that have immunomodulatory (e.g., secretoglobins, cytokines) and protective (e.g., trefoil proteins, heregulin) functions [4–8]. Moreover, a lower expression of toll-like receptors (TLRs) on epithelial cell membranes has been reported, and this leads to less efficient recognition of pathogens and a delayed and less effective induction of the innate immune response [9, 10]. On the other hand, the activity of lymphocytes, macrophages, and dendritic cells is poor in the first years of life and tends to reach adult efficiency only after repeated exposure to infectious agents. The secretion of pro-inflammatory cytokines and chemokines is low for the first years of life, as is the production of serum and mucosal antibodies [11–14].

Several attempts to improve immune function in young children have been made using alternative medicines (e.g., plant preparations), dietary supplements (e.g., vitamins C and D, zinc, cod liver oil and polyunsaturated fatty acids), and other preparations such as inosine pranobex, probiotics, bacterial lysates and pidotimod (PDT) [15–17]. In only a few cases, scientific evidence of positive effects was found. However, encouraging results have been collected with PDT, a synthetic dipeptide molecule (3-l-pyroglutamyl-l-thiazolidine-4carboxilic acid) that seems to have immunomodulatory activity on both innate and adaptive responses. Higher expression of TLR2 proteins, induction of dendritic cell maturation accompanied by an increased release of pro-inflammatory molecules, upregulation of the expression of HLA-DR, stimulation of T lymphocyte proliferation and differentiation toward a Th1 phenotype, inhibition of thymocyte cell death and promotion of phagocytosis have all been demonstrated in in vitro studies in both animal and human subjects [18–20]. Moreover, in vivo studies have demonstrated that long-term prophylactic use of PDT can be of benefit in children with RRTIs, reducing the total number of new infectious episodes and the consequent use of drugs, including antibiotics [21]. A recent Italian study showed that PDT treatment 400 mg/day for 2 months was able of significantly reducing the number of children with upper and lower airways symptoms, and medications use, increasing school attendance, and reducing pediatric visits for RRTIs [22]. Finally, it was found that in subjects with Down syndrome, the response to the influenza vaccine administered at the beginning of a 90 day-PDT course was different from the response in untreated children, suggesting a preferential activation of effector mechanisms and a potential beneficial effect of immunization [23].

Until now, the effects of PDT on the immune system have only been studied in vivo after long-term administration to evaluate whether its immunomodulatory activity might prevent the development of infections. No data are available on the immunological impact of PDT when given during an acute disease. Information regarding this could be useful to understand whether this drug could positively influence the clinical course of an acute infection. This study was planned to evaluate the immunomodulatory activity of PDT administered together with standard antibiotic therapy in children hospitalized for community-acquired pneumonia (CAP).

## Methods

### Study design

This study was carried out at the Pediatric Highly Intensive Care Unit of the University of Milan, Fondazione IRCCS Ca’ Granda Ospedale Maggiore Policlinico, Milan, Italy between November 1, 2013 and April 30, 2014. It was approved by the Ethics Committee of the Fondazione IRCCS Ca’ Granda Ospedale Maggiore Policlinico, Milan, Italy. Written informed consent was obtained from the parents or legal guardians of all study participants, and in the case of patients aged ≥7 years, written consent from the children was also collected.

Only otherwise healthy children aged 3–14 years with clinical signs such as tachypnea and abnormal breath sounds and a chest radiograph consistent with non-complicated CAP were considered eligible for the study. Complicated CAP was defined as the presence in a chest radiograph of more than one of the following conditions: parapneumonic effusion, defined as loculated pleural fluid; any pleural fluid parameters consistent with empyema; atelectasis; and necrotizing pneumonia [24, 25]. The CAP diagnoses were all confirmed by chest radiographs evaluated by an independent expert radiologist who classified the findings as alveolar pneumonia, nonalveolar pneumonia or no pneumonia in accordance with the World Health Organization criteria for the standardized interpretation of pediatric chest radiographs for the diagnosis of pneumonia [26].

Upon enrollment, detailed information regarding children’s demographics, clinical history and the characteristics of the disease were collected together with a blood sample for the evaluation of laboratory variables, including white blood cell (WBC) counts, C-reactive protein (CRP) and procalcitonin (PCT) levels. A portion of the blood sample obtained at the time of recruitment (T0) (i.e., before therapy administration) was also used for the immunological analyses described below. The enrolled children were randomly assigned in a 1:1 ratio and according to a computer-generated list to receive either standard antibiotic therapy with cefotaxime (100 mg/kg/day in 3 daily doses, i.v.) plus clarithromycin (15 mg/kg/day in two daily doses, orally) (control group) according to the guidelines for the treatment of pediatric CAP prepared by the Italian Society of Pediatrics [27] or the same antibiotics plus PDT (800 mg/day in two daily doses, orally) (PDT group). In both groups, cefotaxime was administered for 4 days and then followed by amoxicillin-clavulanate (80 mg/kg/day in 3 daily doses, orally, for 6 days), and clarithromycin was administered at the same dosage for 14 days; in the PDT arm, PDT was given at the same dosage for 14 days.

Blood samples for the evaluation of immunological parameters were drawn at T3 and T5 (i.e., 3 and 5 days after the initiation of therapy) as well as at T21 (i.e., 7 days after the therapy ended). All of the information was registered in an electronic database.

### Immunological analyses

Whole blood was collected by venipuncture in Vacutainer tubes containing ethylenediaminetetraacetic acid (EDTA, BD Vacutainer, San Diego, CA, USA). PBMCs were separated from whole blood by density gradient centrifugation on Ficoll (Cedarlane Laboratories Limited, Hornby, Ontario, Canada) after centrifugation for 25 min at 2300 rpm. PBMC layers were carefully removed and washed twice in phosphate-buffered saline (PBS, PBI, Milan, Italy), and cell counts and viability were determined with an automated cell counter (ADAM-MC, Digital Bio, NanoEnTek Inc., Korea).

PBMCs were incubated for 3 or 24 h for mRNA or protein analyses, respectively, in the following conditions: without any stimulus, in the presence of a mixture of 8 pneumococcal polysaccharides (10 μg/mL) (ATCC^®^ Pneumococcal polysaccharide type 23, 4, 14, 9, 57, 6A, 3, 5, LGC Standards, Milan, Italy) or in the presence of lipopolysaccharide (LPS, Sigma-Aldrich, St. Louis, MO, USA) (2 μg/mL). Anti-CD28 antibody (R&D Systems, Minneapolis, MN, USA) was added during incubation (2 μg/mL) to facilitate costimulation. For cytokine analyses, 10 μg/mL brefeldin A (Sigma-Aldrich, St. Louis, MO, USA) was added to the cultures after 3 h of stimulation to block protein secretion.

After 18 h of stimulation, PBMCs were washed, resuspended in PBS (PBI, Milan, Italy), and split into different flow cytometry tubes. Antigen presenting cells (APCs) were evaluated by flow cytometric analyses. PBMCs were stained for 15 min at room temperature for anti-CD14, TLR4, TLR2, CD11c, HLA-DRII, CD80 and CD86.

Intracellular cytokine secretion was assessed with monoclonal antibodies (mAbs) specific for tumor necrosis factor (TNF)-α and interleukin (IL)-12. After 45 min of incubation at 4 °C in the dark, cells were washed and fixed in 1 % paraformaldehyde in PBS.

The following monoclonal antibodies (mAbs) were used: anti-human CD11c (mouse IgG1 isotype) phycoerythrin-Cy7 (PECy7), anti-human CD14 (mouse IgG1 isotype) R-phycoerythrin-cyanine 5 (PECy5), anti-human HLA-DRII (mouse IgG1 isotype) R-phycoerythrin-cyanine 5 (PECy5), anti-human CD86 (mouse IgG1 isotype) phycoerythrin (PE), anti-human TLR2 (mouse IgG2a isotype) and anti-human CD80 all coupled to fluorescein isohiocyanate (FITC) (Beckman-Coulter, Milano, Italia). The mAbs used for detection via intracellular staining were anti-human TNF-α (mouse IgG1 isotype) phycoerythrin (PE) (R&D Systems, Minneapolis, MN, USA) and anti-human IL-12 (mouse IgG1 isotype) fluorescein isohiocyanate (FITC) (BD Biosciences, San Diego, CA).

Cytometric analyses of phenotypes and cytokine secretion were performed using an FC500 flow cytometer (Beckman-Coulter, Milano, Italia) equipped with a double 15-mW argon ion laser with a wavelength of between 456 and 488 nm interfaced with an Intercorp computer. Green fluorescence from FITC (FL1) was collected through a 525 nm band-pass filter; orange-red fluorescence from PE (FL2) was collected through a 575 nm band-pass filter; Texas red fluorescence from ECD (FL3) was collected through a 613 nm band-pass filter; red fluorescence from PECy5 (FL4) was collected through a 670 nm band-pass filter; far red fluorescence from PECy7 (FL5) was collected through a 770 nm band-pass filter. Data were collected using linear amplifiers for forward and side scatter and logarithmic amplifiers for FL1, FL2, FL3, FL4 and FL5. Samples were first run using isotype controls or single fluorochrome-stained preparations for color compensation. For each analysis, 20,000 events were acquired and gated for CD14 and CD11c expression and SSC properties.

After 3 h of pneumococcal polysaccharide stimulation, RNA was extracted from PBMCs by the acid guanidinium thiocyanate-phenol–chloroform method. The RNA was dissolved in RNase-free water and purified from genomic DNA with RNase-free DNase (RQ1 DNase, Promega, Madison, Wisconsin, USA). One microgram of RNA was reverse transcribed into first-strand cDNA in a 20-µL final volume containing 1 µM random hexanucleotide primers, 1 µM oligo dT and 200 U Moloney murine leukemia virus reverse transcriptase (Clontech, Palo Alto, California, USA).

The antibacterial response signaling pathway was analyzed using a real-time polymerase chain reaction PCR array including a set of optimized real-time PCR primer assays on 96-well plates (SABiosciences Corporation, Frederick, MD, USA). This approach permits the monitoring of the mRNA expression of 84 genes involved in the innate immune response to bacteria plus five housekeeping genes. The procedures were carried out according to the manufacturer’s suggestions. Experiments were run on samples from all the subjects included in the study pooled into two groups: PDT and control. Thus, the results represent the mean values of the different targets analyzed in the PDT and control subjects. The results were analyzed with SABiosciences online software, calculated relative to the housekeeping genes and expressed as ΔΔCt. Only targets that exhibited at least a 2-fold modulation were considered significant.

### Statistical analysis

In the tables displaying the main and clinical characteristics at baseline, continuous variables are presented as mean values and standard deviations (SDs) and were analyzed using a non-parametric Wilcoxon-Mann–Whitney test. Categorical variables are presented as numbers and percentages and were analyzed by the Fisher’s exact test. All the tests were two-sided, and a p value <0.05 was considered statistically significant.

For the immunological data, p values for the differences between the PDT and control groups at each time point (i.e., day 3, day 5 and day 21) were estimated via two-tailed Wilcoxon-Mann–Whitney tests. The p values for the differences between the PDT and control groups across time were estimated via a 2-way ANOVA on ranks. We also performed a test to evaluate the linear trend between the PDT and control groups across time using a 2-way ANOVA on ranks with a first degree polynomial contrast for time. The data were analyzed using SAS version 9.1 statistical software (SAS Institute, Cary, NC, USA).

## Results

### Study population

Table 1 shows the demographic and clinical characteristics of the study patients. A total of 20 children (mean age ± SD, 4.6 ± 1.9 years; 14 [70.0 %] males) hospitalized for CAP were enrolled. Ten received PDT plus standard antibiotic therapy and 10 received standard antibiotics only. The two groups were comparable for all the studied demographic and health history variables, including ethnicity, parental smoking habits, exclusive breastfeeding for ≥3 months, previous diagnosis of allergic disease, and respiratory infection or antibiotic treatment in the previous 6 months. Clinical and laboratory characteristics of the study children were also similar between the groups. In particular, no significant differences at baseline were found between children randomized to receive PDT plus antibiotics and those who received antibiotics only with regard to the prevalence of fever, cough, or dyspnea, or with regard to thoracic findings, WBC counts, or CRP and PCT levels.

**Table 1:** Main demographic, clinical and laboratory characteristics at baseline in 20 children hospitalized for community-acquired pneumonia according to the use of pidotimod plus antibiotics or antibiotics only

Characteristic	All children (n = 20)	Pidotimod + antibiotics (n = 10)	Antibiotics only (n = 10)	p-value
Demographics				
Age (years)				
Mean (SD)	4.6 (1.9)	4.4 (2.0)	4.7 (1.8)	0.70^a^
Sex (%)				
Male	14 (70)	7 (70)	7 (70)	
Female	6 (30)	3 (30)	3 (30)	1
Ethnicity (%)				
Caucasian	17 (85)	8 (80)	9 (90)	
Non-caucasian	3 (15)	2 (20)	1 (10)	0.99^b^
Parental smoking habit^b^ (%)				
Both non-smokers (%)	13 (65)	6 (60)	7 (70)	
At least one smoker (%)	7 (35)	4 (40)	3 (30)	0.99^b^
Exclusive breastfeeding ≥3 months^c^				
Yes (%)	11 (58)	4 (40)	7 (78)	
No (%)	8 (42)	6 (60)	2 (22)	0.17^b^
Diagnosis of allergy (%)				
Yes	18 (90)	9 (90)	9 (90)	
No	2 (10)	1 (10)	1 (10)	1
Respiratory infections in the previous 6 months (%)				
At least one	13 (65)	5 (50)	8 (80)	
None	7 (35)	5 (50)	2 (20)	0.35^b^
Use of antibiotics for respiratory infection in the previous 6 months (%)				
Yes	4(20)	2(20)	2(20)	
No	16 (80)	8 (80)	8 (80)	1
Clinical characteristics				
Fever (≥37.8°)^c^ (%)				
Yes	8 (42)	5 (56)	3 (30)	0.37^b^
No	11 (58)	4 (44)	7 (70)	
Cough^c^ (%)				
Yes	18 (95)	8 (89)	10 (100)	
No	1 (5)	1 (11)	0 (0)	0.47^b^
Dyspnea^c^ (%)				
Yes	4 (21)	2 (22)	2 (20)	
No	15 (79)	7 (78)	8 (80)	0.99^b^
Rhonchi (%)				
Yes	1 (5)	1 (10)	0 (0)	
No	19 (95)	9 (90)	10 (100)	0.99^b^
Rales (%)				
Yes	16 (80)	7 (70)	9 (90)	
No	4 (20)	3 (30)	1 (10)	0.58^b^
Wheezes (%)				
Yes	4 (20)	3 (30)	1 (10)	
No	16 (80)	7 (70)	9 (90)	0.58^b^
SpO_2_ <92 % (%)				
Yes	7 (35)	2 (20)	5 (50)	
No	13 (65)	8 (80)	5 (50)	0.35^b^
Laboratory results				
White blood cell count (cells/µL)				
Mean (SD)	13,154 (6512)	13,176 (6708)	13,132 (6672)	0.97^a^
CRP (mg/L)				
Mean (SD)	5.95 (6.58)	5.82 (5.02)	6.07 (4.25)	0.82^a^
PCT (ng/mL)				
Mean (SD)	0.40 (0.84)	0.42 (0.89)	0.38 (0.84)	0.96^a^

*CRP* C reactive protein, *PCT* procalcitonin, *SD* standard deviation

^a^p value from Wilcoxon-Mann–Whitney test

^b^p value from Fisher’s Exact Test

^c^The sum does not add up to the total because of missing values

### PDT induces dendritic cell maturation and activation

**Fig. 1:**
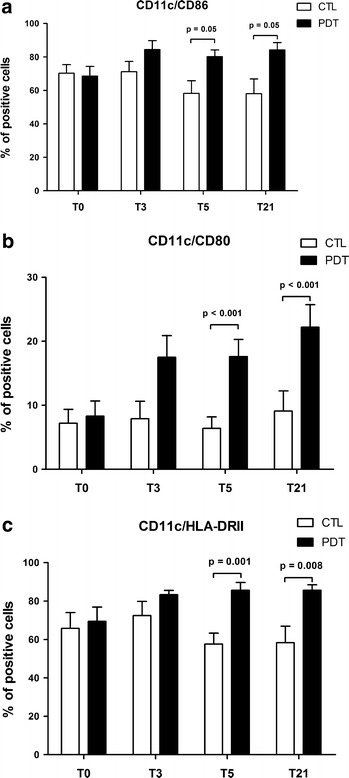
Percentages of pneumococcal-stimulated positive dendritic cells (CD11c^+^) expressing HLA-DRII (**a**), CD86 (**b**) and CD80 (**c**) molecules are shown at baseline and in response to therapy in children with community-acquired pneumonia (CAP) treated with antibiotics plus pidotimod and in controls treated with antibiotics only. For each analysis, 30,000 events were acquired and gated on CD11c expression and side scatter properties. Mean values + SD and statistically significant differences are indicated

The data regarding dendritic cell (DC) maturation according to treatment arm are reported in Fig. 1. DCs were characterized by analyzing the expression of surface markers associated with cell activation and maturation, MHC class II molecules (HLA-DRII) and costimulatory molecules (CD80, CD86). Cytokine production was assessed upon PBMC stimulation in vitro with pneumococcal polysaccharides or LPS. The surface and morphological activation status of DCs was comparable at baseline in both groups of subjects. The percentage of CD11c^+^ cells expressing either HLA-DRII (Fig. 1a), CD80 (Fig. 1b) or CD86 (Fig. 1c) significantly increased in the PDT group compared to the controls 5 and 21 days after therapy initiation. The difference in CD80 expression was also significant across time (p for trend = 0.041). Similar results were obtained upon stimulation with LPS (data not shown).

**Fig. 2:**
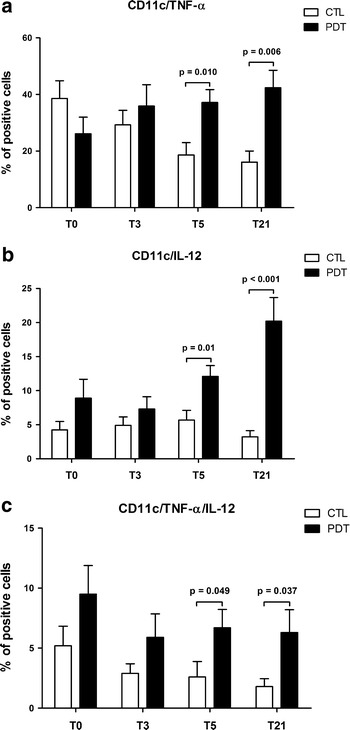
Percentages of pneumococcal-stimulated dendritic cells (CD11c^+^) secreting TNF-α (**a**) and IL-12 (**b**) are shown at baseline and in response to therapy in children with community-acquired pneumonia (CAP) treated with antibiotics plus pidotimod and in controls treated with antibiotics only. The percentage of TNF-α/IL-12 secreting CD11c^+^ cells was higher in the therapy group than in the controls, although significant differences were observed only at T5 and T21 (**c**). For each analysis, 30,000 events were acquired and gated on CD11c expression and side scatter properties. Mean values + SD and statistically significant differences are indicated

Analyses of cytokine release from DCs revealed that the percentage of CD11c^+^ cells secreting TNF-α or IL-12 became higher in the PDT group compared to the control group at days 5 and 21, with a significant p for trend for both cytokines across time (p = 0.007, and p = 0.030, respectively) (Fig. 2a, b). The percentage of CD11c^+^ cells secreting TNF-α or IL-12 was higher at all times, although not significantly, in the PDT group than in the controls (Fig. 2c). Similar data were observed when cells were stimulated with LPS (data not shown).

### PDT induces monocyte maturation and activation

**Fig. 3:**
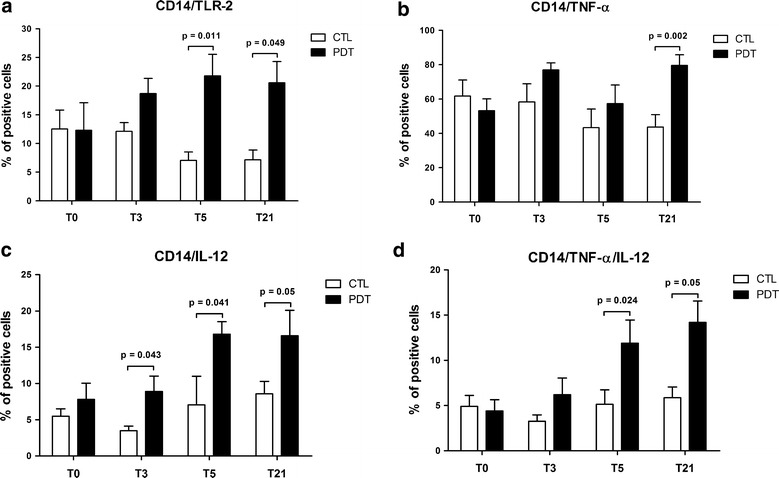
TLR2 expression on monocytes (CD14^+^) (**a**) and percentages of CD14 + cells expressing TNF-α (**b**), IL-12 (**c**) or TNF-α/IL-12 (**d**) are shown at baseline and in response to therapy in children with community-acquired pneumonia (CAP) treated with antibiotics plus pidotimod and in controls treated with antibiotics only. For each analysis, 30,000 events were acquired and gated on CD11c expression and side scatter properties. Mean values + SD and statistically significant differences are indicated

Activation of monocytes (CD14^+^ cells) via TLRs and secretion of pro-inflammatory mediators was also evaluated after stimulation of PBMCs with pneumococcal polysaccharides or LPS. As shown in Fig. 3a, TLR2 expression on CD14 + cells was significantly higher in the PDT group compared with the control group 5 and 21 days after treatment initiation, with a significant p for trend for the comparison across time between treatment groups (p = 0.023). Peak inflammatory responses were detected 5 to 21 days after therapy administration with the strongest effect observed in either TNF-α-, IL-12- or TNF-α/IL-12-secreting CD14 + cells in the PDT group compared with the control subjects (Fig. 3b–d).

### PDT upregulates antibacterial responses

**Fig. 4:**
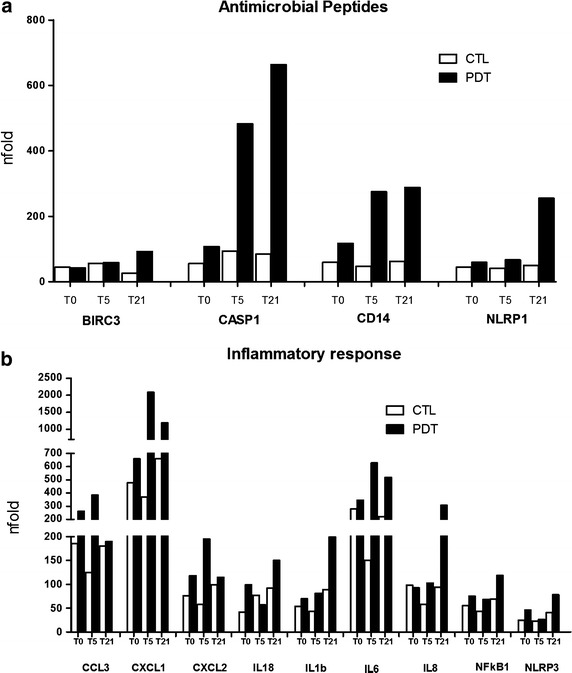
Antibacterial response signaling pathway: antimicrobial peptides (**a**) and genes involved in the inflammatory response (**b**) at baseline as well as at days 3, 5 and 21 in children with community-acquired pneumonia (CAP) treated with antibiotics plus pidotimod and in controls treated with antibiotics only

Figure 4 summarizes gene expression related to the antibacterial response signaling pathway (Panel a). Following pneumococcal polysaccharide stimulation, mRNA expression of antimicrobial peptides reached a peak of expression at day 5 with a subsequent decrease at day 21. The results demonstrate the same trend in both groups of patients, but the increases were more pronounced in the PDT-treated individuals. Likewise, an upregulation of inflammatory response genes was also observed in the PDT-treated group in comparison with the control group (Panel b).

## Discussion

This is the first study in which the influence of PDT on immune system function during an acute infectious disease was evaluated. Our data indicate that PDT administration in addition to standard antibiotic therapy in children with CAP may significantly increase the natural immune system response to an infectious stimulus via a direct influence on DC maturation and function, TLR2 expression in monocytes, antimicrobial peptide secretion and upregulation of genes involved in the inflammatory response. The positive effect was observable after only a few days of PDT administration and remained evident for several days after the drug treatment ended.

DCs are cells of the mononuclear phagocyte system that play a critical role in the regulation of the adaptive immune response [28]. At steady state, DCs are described as immature, a phenotype characterized by low surface expression of MHC class II molecules and co-stimulatory molecules such as CD80 and CD86. In response to activation by infection, DCs undergo a program of maturation that leads to the acquisition of a number of fundamental properties including antigen processing and presentation, migration and T cell co-stimulation. In this context, upregulation of co-stimulatory molecules and HLA-DRII antigens is evidenced together with a significant production of pro-inflammatory cytokines, such as IL-12 and TNF-α, which play an important role in the polarization of T helper cell subsets toward a Th1 profile during priming by DCs and thus experience the functional consequences of DC activation [29].

In this study, it was found that PDT administration results in DC activation and maturation as evidenced by the increased number of DCs expressing costimulatory molecules such as CD86 and CD80. Stimulation of the immune system induced by PDT is also evidenced by the increase in the number of cells that, particularly after exposure to bacterial components, were found to be able to produce IL-12 as well as TNF-α. Similar results were observed regarding TLR expression and proinflammatory cytokine production in monocytes. In particular, TLR2 plays a major role in promoting protective immunity against respiratory pathogens [30], and its upregulation could reduce susceptibility to further respiratory infections. In contrast in PDT-treated children, the increased expression of antimicrobial peptides (e.g., CAMP, LCN2, LTF and MPO) and genes involved in cell chemotaxis, the upregulation of the inflammatory response and increased apoptosis [31–33], (e.g., CCL3, CXCL1, CXCL2, IL-18, IL-1b, IL-6, IL-8, NFkB1, and NLRP3) suggest that PDT administration might significantly increase the activity of the immune system for a long period of time, thus reducing the risk of early recurrences during CAP in children.

Our study was a pilot project with an immunological primary endpoint; to observe clinically significant differences during the treatment of an acute pediatric infection, a larger sample size is needed. Our results encourage further studies on infected mice in order to confirm these immunological findings and their clinical relevance as well as on larger populations using PDT in addition to the current standard of care for the acute treatment of pediatric CAP in order to evaluate whether the observed biological effects are associated with differences in the clinical outcome. In addition, further research should evaluate how long this positive immune effect persists and whether it is also observed in complicated CAP cases and other pediatric infectious diseases.

## Conclusions

This study demonstrates for the first time that PDT administration together with standard antibiotic therapy is associated with a favorable persistent immunomodulatory effect in children with CAP, suggesting that it could reduce the risk of early recurrences.

## Abbreviations

CAP: community-acquired pneumonia
CRP: C-reactive protein
DC: dendritic cell
IL-12: interleukin-12
mAbs: monoclonal antibodies
PCR: polymerase chain reaction
PCT: procalcitonin
PDT: pidotimod
RRTIs: recurrent respiratory tract infections
SD: standard deviation
TLRs: toll-like receptors
TNF-α: tumor necrosis factor-α
WBC: white blood cell counts
